# Prediction of risk factors of bronchial mucus plugs in children with *Mycoplasma pneumoniae* pneumonia

**DOI:** 10.1186/s12879-021-05765-w

**Published:** 2021-01-13

**Authors:** Jiahui Zhang, Ting Wang, Rongrong Li, Wei Ji, Yongdong Yan, Zhichao Sun, Jiahong Tan, Jinfeng Wu, Li Huang, Zhengrong Chen

**Affiliations:** Department of Respiratory Medicine, Children’s Hospital of Soochow University, Suzhou, 215003 China

**Keywords:** *Mycoplasma pneumoniae* pneumonia, Children, Mucus plug, Risk factors

## Abstract

**Background:**

Recently, many cases of pneumonia in children with *Mycoplasma pneumoniae* infection have been shown to have varying degrees of intrabronchial mucus plug formation. The clinical, laboratory, radiological characteristics, and treatment of patients with Mycoplasma infection are analyzed in this study. The risk factors for *M. pneumoniae* pneumonia (MPP) mucus plug formation in children are explored, and a risk factor scoring system is established.

**Methods:**

MPP patients treated with bronchoscopy were retrospectively enrolled in the study from February 2015 to December 2019. The children were divided into a mucus plug group and a control group according to the presence or absence of mucus plug formation. The clinical, laboratory, radiological characteristics, and treatment of the two groups of children were compared. Univariate and multivariate logistic regression models were used to identify the risk factors for MPP mucus plug formation. The receiver operating characteristic (ROC) curve was drawn to evaluate the regression model and establish the MPP mucous plug risk factor scoring system.

**Results:**

A univariate analysis showed that the children in the mucous group were older and had a longer fever duration, longer hospital stay, higher fever peak, more cases of wheezing symptoms and allergies, and azithromycin or corticosteroids were administered later. In addition, neutrophil, C-reactive protein (CRP), lactate dehydrogenase (LDH), D-dimer (DD), sputum MP-DNA copy number, and total immunoglobulin A (IgA) levels were higher, while prealbumin (PA) levels were lower. The ROC curve analysis showed that children with MPP had PA ≤144.5 mg/L, had used corticosteroids during the course of the illness of ≥4.5 days, CRP ≥12.27 mg/L, an LDH ≥ 462.65 U/L, and there was a possibility of intra-airway mucus formation. The independent risk factors were scored according to their odds ratio (OR) value. Among the 255 children with MPP, the high-risk group had 44 (83.02%) mucus plugs out of 53; the middle-risk group had 35 (34.3%) mucus plugs out of 102; and the low-risk group had 11 (11%) mucus plugs out of 100.

**Conclusions:**

PA levels, timing of corticosteroid use (use in the first few days), CRP levels, and LDH levels were independent risk factors for MPP mucus plug formation. This provides a basis for the early identification of MPP in children combined with mucus plug formation.

## Background

*M. pneumoniae* is an important and common pathogen in respiratory infections in children [1, 2]. *M. pneumoniae* pneumonia (MPP) accounted for 34.75% of community-acquired pneumonia (CAP) in hospitalized children from the Children’s Hospital of Soochow University from January 2011 to December 2015, with children over 5 years of age being more commonly affected than younger children [3–6]. The acute phase of MPP can present with varying degrees of damage to the airway mucosa, which in severe cases can lead to mucosal embolization of the orifice and inflammatory stenosis or even occlusion. In addition, more than 30% of refractory MPP have been found to form bronchial mucus plugs (BMPs) [7].

BMPs are endogenous bronchial foreign bodies that are caused by inflammation, bleeding, necrosis, abnormal secretion of bronchial mucus in the bronchus, mucus elimination obstacles, and then mucus accumulation and agglomeration in the bronchus, forming bronchial mucus plugging [8, 9]. If not cleared in time, they can lead to bronchodilatation, pulmonary arrhythmia, occlusive bronchitis, and even acute respiratory failure and the blockage can be seriously life-threatening.

A study by Xu Q et al. showed that in patients aged 5 years and older, higher IL-10 levels and higher IFN-γ levels had an important predictive value for mucus plug formation [10]. In the predicted nomogram, atelectasis and pleural effusion had the highest score and the highest weight, which is a powerful indicator for bronchoscopy intervention [11]. The aim of this study was to analyze the risk factors for the formation of BMP in children with MPP. This evaluation of risk factors can assist clinicians to judge whether there is a possibility of BMP formation and ascertain the opportunity for reasonable treatment. Thus, it has important significance for reducing the occurrence of irreversible damage.

## Materials and methods

### Patients and data collection

This retrospective study was conducted in the Children’s Hospital of Soochow University. A total of 255 children who met the diagnosis of MPP were selected and treated with bronchoscopy during hospitalization from February 2015 to December 2019. The age range of the selected 255 children with MPP ranged from 2 months to 16 years. They all met the diagnostic criteria of the MPP diagnosis and treatment expert consensus (2015 version) for children. The clinical manifestations were fever, cough, and dyspnea, and with or without other systemic manifestations, such as dry lungs and wet rales, with signs of pulmonary consolidation and changes in lung imaging. In addition, the conditions that needed to be met were a serum MP-IgM > 1.1 or nasopharyngeal aspirates (NPA) MP-DNA > 1.0 × 105 copies/L. The exclusion criteria included those patients with chronic lung disease, recurrent respiratory tract infections, recurrent wheezing or a medical history of asthma, bronchopulmonary dysplasia, immunosuppression or defective disease, severe heart, liver, kidney disease, malignant tumors, and incomplete case information.

Laboratory tests were completed within 24 h after the children were admitted to the hospital, including neutrophil, CRP, LDH, DD, MP-IgM, PA levels, and NPAs. Demographic and clinical data of 255 children with MPP, including epidemiological, clinical, laboratory, and radiological characteristics, and bronchoscopy results, as well as treatments and outcomes, were collected and recorded.

### Fiberoptic bronchoscopy

The children were fasted for 4–6 h prior to surgery. Atropine (0.01–0.02 mg/kg) and midazolam (0.1–0.3 mg/kg, the maximum amount was 4 mg/time) were injected intramuscularly 30 min prior to surgery. A solution of 2% lidocaine was swallowed nasally and orally three to four times, and 1% rosemary nasal drops were administered to the right nose. The choice of bronchoscopy model depended on patient age. The bronchoscope reached the openings of the trachea and the left and right bronchi through the nose and epiglottis. The front end of the bronchoscope reached the lesion and was then embedded in the lumen. Bronchial alveolar lavage was performed using 0.9% saline at 37 °C. For local lavage, where it was difficult to remove the mucus tie, it was removed with a brush or biopsy forceps and slowly pulled out of the fiberoptic bronchoscope.

### Definitions

According to the performance of bronchoscopy, patients were divided into a mucous plug group and a control group. In the mucus group, the sputum plugs could be seen in the bronchial cavity of the lungs that blocked the lumen, and some plastic sputum plugs had formed. These are not easily removed and require the use of a brush. Some even require the use of foreign body pliers. In the control group, there were no mucus plugs in the lumen of the bronchus under the bronchoscope, but a few flocculent or thin secretions in the lumen could be seen.

### Statistical analysis

SPSS25.0 statistical software was used for the data analysis. Measurement data conforming to a normal distribution are expressed as means ± standard deviations (x ± s). The comparison between the two groups used an independent sample t test. Non-normal distribution data is expressed as the median, and the comparison between the two groups used the Wilcoxon rank sum test. A *P* < 0.05 was considered statistically significant. The count data is expressed as a percentage (%), and a comparison between the groups was performed using a χ^2^ test. A logistic regression analysis of the risk factors related to intratracheal mucus plug formation in children with MPP was also performed (variable selection criteria were *P* < 0.05 and elimination criteria were *P* > 0.1; test level was bilateral α = 0.05). The receiver operating characteristic (ROC) curve was drawn, and the area under curve (AUC) was used to evaluate the predictive value of each independent risk factor in the formation of mucus plugs. A *P* < 0.05 was considered statistically significant.

## Results

### Clinical characteristics

**Fig. 1:**
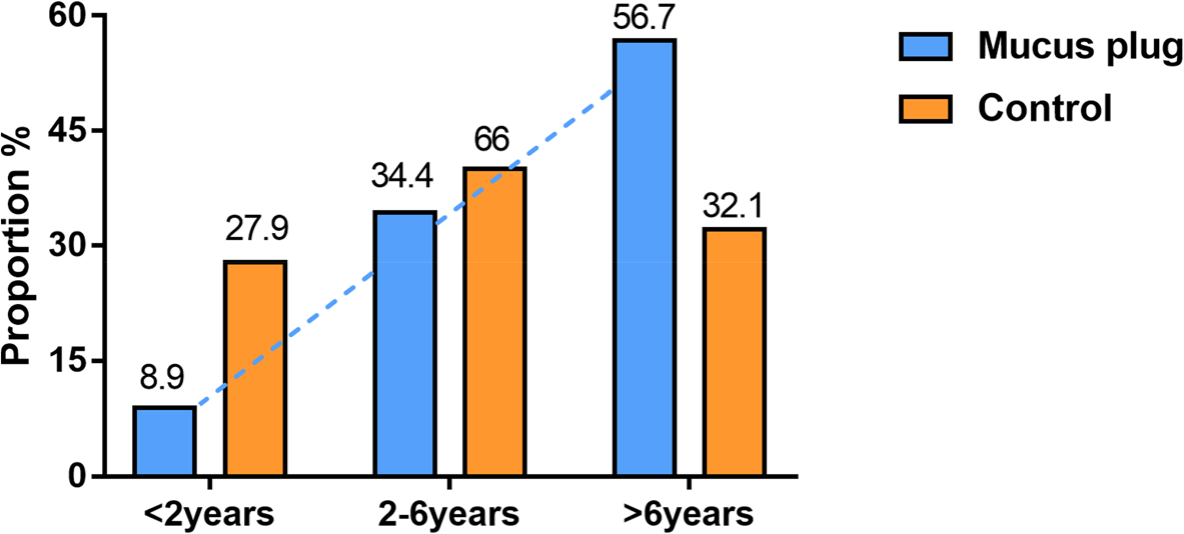
Age distribution at onset

**Fig. 2:**
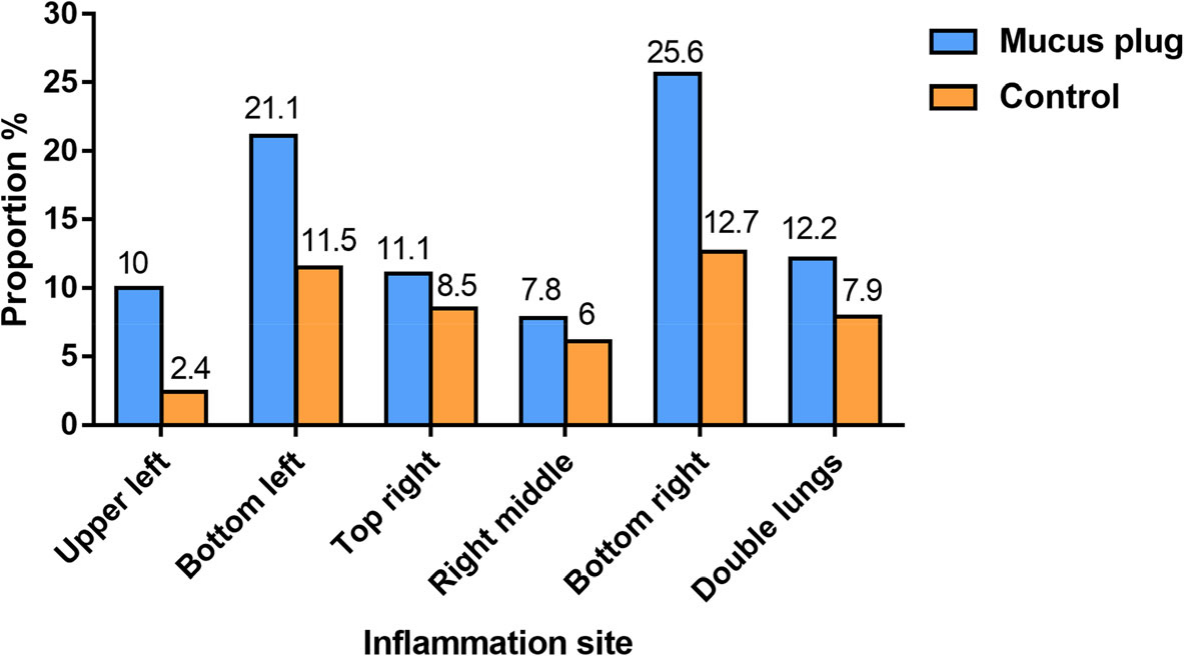
Inflammation site distribution

**Fig. 3:**
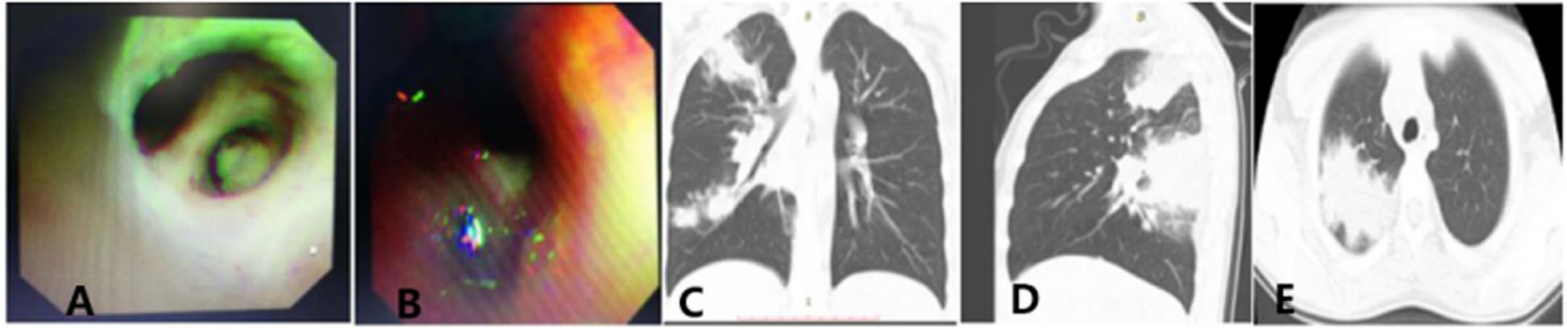
This is a bronchoscopic performance and imaging features of a MPP patient. Bronchoscopy performance (A and B): A is the plastic sputum plug of the upper right and the posterior branch. The wall of the tube is smooth after the brush removes it; B is the shaped sputum plug on the left side of B8 and B9, and the lumen is smooth after lavage; C and D are the normal and lateral pictures of this child’s chest radiograph; and E is a CT slice of this child. CT = computed tomography; MPP = *M. pneumoniae* pneumonia

There were 255 children with MPP who were treated with bronchoscopy. The mucus plug group accounted for 90 (35.3%) cases and the control group for 165 (64.7%) cases. The mean ages of the mucus plug and control group patients were 6.10 ± 2.85 years and 4.50 ± 2.93 years, respectively, with a significant difference (*P* < 0.05). The males in the mucus plug group and the control group were 49 (54.4%) of 90 and 89 (53.9%) of 165, respectively, and the difference was not statistically significant (*P* > 0.05; Table 1). In the mucus plug group, children < 2 years of age were significantly less than 2–6 years old and > 6 years old, with children > 6 years old being the majority, and the difference was statistically significant (*P* < 0.017; Fig. 1). The univariate analysis showed that the children in the mucus plug group were older, had a longer fever duration, longer hospital stay, higher fever peak, more cases of fever, wheezing symptoms and allergies, azithromycin (intravenous or oral) or corticosteroids (intravenous or oral) were administered later, and the neutrophil, C-reactive protein (CRP), lactate dehydrogenase (LDH), D-dimer (DD), sputum MP-DNA copy number, total IgA, and the CD19 + CD23+ levels were higher, while the PA levels were lower. In addition, lung consolidation and pleural effusion cases were greater (Table 1). The chest radiograph or computed tomography (CT) showed that the lung inflammation in the mucous group was primarily in the bottom right lung (23 [25.6%] of 90) and the bottom left lung (19 [21.1%]; Fig. 2). Figure 3 is a bronchoscopic performance and imaging features of a 10-year-old MPP patient.

**Table 1:** Clinical characteristics of patients in the mucus plug and control group

Variables	Mucus plug group (*n* = 90)	Control group (*n* = 165)	*P* value
**Characteristics**			
Gender (male/female, n)	49/41	89/76	0.938
Age (^−^x ± s)/year	6.10 ± 2.85	4.50 ± 2.93	< 0.01
Length of hospitalization (^−^x ± s) /d	10.72 ± 3.47	8.81 ± 2.72	< 0.01
Course before admission [M(P25–P75)]/d	8 (6, 10)	7 (5, 11)	0.869
Allergic constitution [n (%)]	40 (44.4)	33 (21.2)	< 0.01
**Signs and symptoms**			
Fever [n (%)]	86 (95.6)	135 (81.8)	0.002
Heat range (^−^x ± s) /d	9.72 ± 3.80	5.00 ± 3.98	< 0.01
Hot peak [M(P25–P75), °C]	39.6 (39, 40)	39 (38.5, 39.7)	< 0.01
Shortness of breath [n (%)]	6 (6.7)	8 (4.8)	0.542
Breather [n (%)]	7 (7.8)	41 (24.8)	0.01
Lung rales [n (%)]	40 (44.4)	68 (41.2)	0.618
Lung wheezing [n (%)]	6 (6.6)	27 (16.3)	0.27
Reduced breath sounds [n (%)]	14 (15.6)	21 (1.3)	0.531
**Laboratory characteristics**			
WBC [M(P25–P75)]/ × 10^9^ L^−1^	7.63 (5.97, 11.35)	7.97 (6.12, 10.64)	0.844
Neutrophil (^−^x ± s)/%	67.34 ± 12.78	57.53 ± 15.20	< 0.01
Eos [M(P25–P75)]/×10^9^ L^− 1^	0.4 (0.045, 1.325)	0.3 (0.07, 1.5)	0.866
CRP [M(P25–P75)]/mg·L^−1^	25.72 (12.62, 51.23)	7.58 (2.58, 18.23)	< 0.01
PLT [M(P25–P75)]/×10^9^ L^−1^	275.5 (231.75, 356)	321 (232, 394)	0.109
LDH [M(P25–P75)]/U·L^−1^	545.9 (397.3, 655.5)	389.3 (340.0, 389.3)	< 0.01
PA [M(P25–P75)]/mg·L^−1^	114 (92.75, 134)	142 (115, 195.5)	< 0.01
DD [M(P25–P75)]/μg·L^−1^	1207 (484.5, 3677.75)	367 (187, 367)	< 0.01
CK-MB [M(P25–P75)]/ng·mL^−1^	0.9 (0.5, 1.5)	1.1 (0.6, 1.75)	0.295
ALT [M(P25–P75)]/U·L^−1^	17.6 (12.55, 30.725)	12.9 (10.1, 17.8)	< 0.01
AST [M(P25–P75)]/U·L^−1^	34.6 (27.425, 43.725)	30.2 (24.95, 37.95)	0.003
Serum MP-IgM	3.46 (1.36, 5.49)	3.2 (1.15, 5.14)	0.154
Sputum MP-DNA copy number [n (%)]			
Low load group	3 (3.3)	20 (12.1)	0.019
Medium load group	13 (14.4)	42 (25.5)	0.041
High load group	74 (82.2)	103 (62.4)	0.001
Humoral immunity			
IgG (^−^x ± s)/g·L^−1^	9.79 ± 2.86	9.43 ± 3.08	0.357
IgA [M(P25–P75)]/g·L^−1^	1.27 (0.96, 1.76)	1.04 (0.6, 1.53)	0.007
IgM [M(P25–P75)]/g·L^−1^	1.49 (1.09, 2.20)	1.37 (0.95, 1.92)	0.061
Cellular immunity [M(P25–P75), %]			
CD3+	66.96 (61.2, 73.05)	65.5 (60.05, 72.15)	0.634
CD3 + CD4+	34.14 (28.3, 40.32)	34.2 (30.25, 39.2)	0.524
CD3 + CD8+	27.25 (21.5, 27.25)	25.8 (21.5, 30.2)	0.24
CD4+/CD8+	1.30 (1.0, 1.3)	1.3 (1.1, 1.7)	0.117
CD3-CD (15 + 56) +	10.35 (5.7, 15.7)	10.2 (7.1, 14.8)	0.715
CD3-CD19+	19.2 (14.02, 25.92)	18.7 (12.95, 26.85)	0.983
CD19 + CD23+	7.9 (5.4, 12.72)	9.4 (6.55, 13.6)	0.023
**Radiological characteristics [n (%)]**			
Lung consolidation	84 (93.3)	126 (76.36)	0.001
Atelectasis	9 (10)	8 (4.8)	0.123
Pleural effusion	19 (21.1)	18 (10.9)	0.040
Pleural effusion site [n (%)]			
Left side	9 (10)	10 (6.1)	0.331
Right side	10 (11.1)	11 (6.7)	0.238
**Treatment**			
Medication time [M(P25–P75)]/Day n of course			
Azithromycin	5.5 (4, 7)	4 (3, 6)	0.012
corticosteroids	7 (5, 9)	5 (3, 8)	0.003
Fog time before tracheoscopy[M(P25–P75)]/d	3 (2, 4)	3 (2, 5)	0.525

Sputum MP-DNA copy number: low load group, < 1 × 10^4^ L^−1^; medium load group, 1 × 10^4^ L^−1^ –10^6^ L^−1^; high load group, > 1 × 10^6^ L^−1^

*ALT* alanine aminotransferase; *AST* aspartate aminotransferase; *CD* cluster of differentiation; *CK-MB* creatine kinase-MB; *CRP* C reactive protein; *DD* D-dimer; *IgA* immunoglobulin A; *IgG* immunoglobulin G; *LDH* lactate dehydrogenase; *MP Mycoplasma pneumonia; PA* Prealbumin; *PLT* Platelets; *WBC* white blood cells

### Analysis of risk factors for BMP formation in children with MPP

**Fig. 4:**
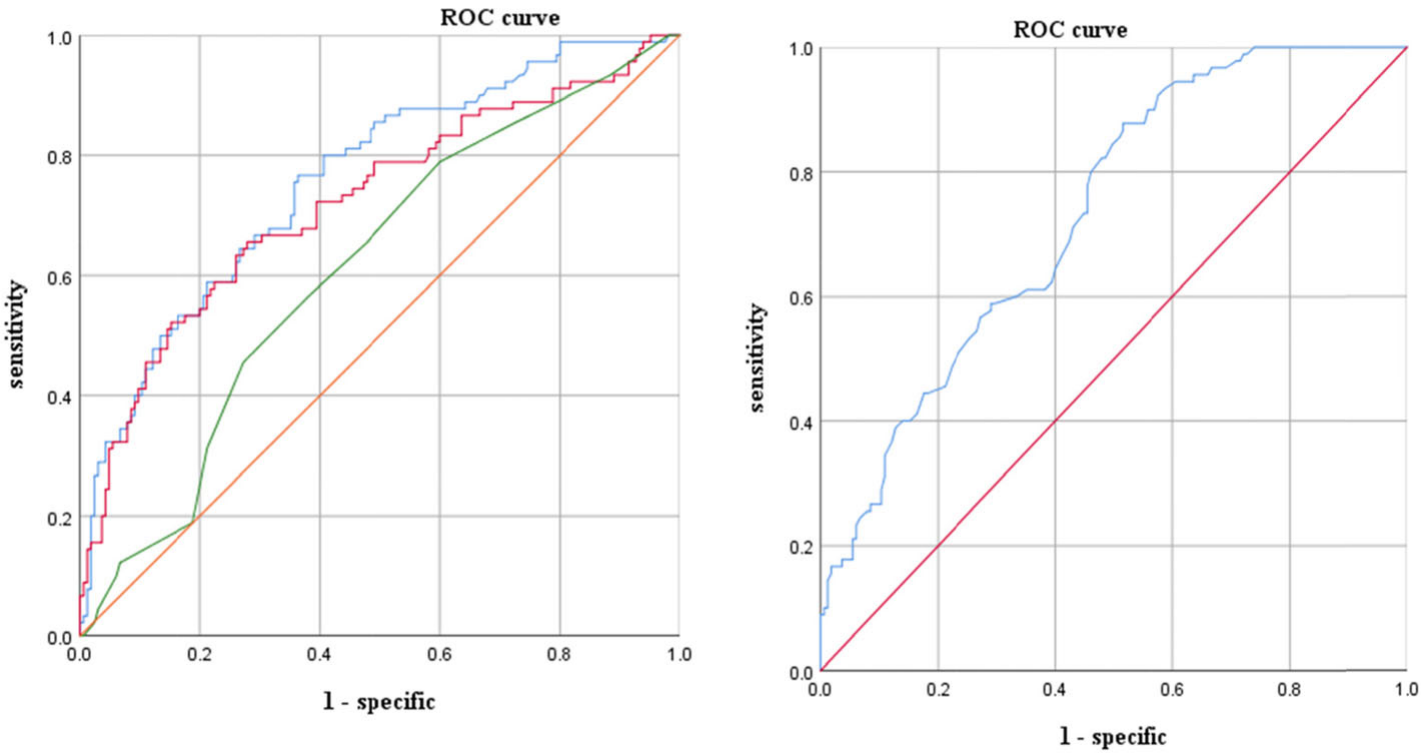
**a** is the ROC curve analysis of the CRP (blue line), LDH (red line), and the timing of corticosteroids application (green line), which predicts the sensitivity and specificity of MPP BMP formation at 76.7 and 63.6%, 65.6 and 72.1%, and 78.9 and 40%, respectively. **b** is the ROC curve analysis of the PA, which predicts that the sensitivity and specificity of MPP BMP formation at 87.8 and 48.5%, respectively. BMP = bronchial mucus plugs; CRP = C reactive protein; LDH = lactate dehydrogenase; MPP = *Mycoplasma pneumoniae* pneumonia; PA = prealbumin; ROC = receiver operating characteristic

A univariate logistic regression showed that the PA level (odds ratio [OR], 1.015; 95% confidence interval [CI], 1.001–1.030), timing of corticosteroids use (use in the first few days; OR, 0.802; 95% CI, 0.663–0.970), CRP level (OR, 0.986; 95% CI, 0.944–0.992), and LDH level (OR, 0.996; 95% CI, 0.992–0.999) were independent risk factors for MPP mucus plug formation (*P* < 0.05, Table 2). The ROC curve analysis showed that when the optimal thresholds for PA, use of corticosteroids during the course, CRP, and LDH were ≤ 144.5 mg/L, ≥4.5 days, ≥12.27 mg/L, and ≥ 462.65 U/L, respectively, their sensitivity and specificity to predict BMP formation were 87.8 and 48.5%, 78.9 and 40%, 76.7 and 63.6%, and 65.6 and 72.1%, respectively (Fig. 4).

**Table 2:** Logistic regression analysis of risk factors related to BMP formation in MPP

Variable	Partial regression coefficient (β)	SE	Wald χ^2^ value	P value	OR (95% CI)
CRP (mg/L)	−0.033	0.013	6.604	0.01	0.986 (0.944–0.992)
LDH (U/L)	−0.004	0.002	5.419	0.02	0.996 (0.992–0.999)
PA (mg/L)	0.015	0.007	4.655	0.031	1.015 (1.001–1.030)
Corticosteroids (d)	−0.220	0.097	5.151	0.023	0.802 (0.663–0.970)
Constant	14.063	6.451	4.753	0.029	–

Corticosteroids, use corticosteroids for the first few days

*BMP* bronchial mucus plug; *CI* confidence interval; *CRP* C reactive protein; *LDH* lactate dehydrogenase; *MPP . pneumoniae* pneumonia; *OR* odds ratio; *PA* prealbumin; *SE* standard error

### Logistic regression analysis after assigning risk factors for BMP formation

The critical values of the independent factors were assigned to evaluate the risk of BMP formation. The logistic regression analysis showed a statistical significance (*P* < 0.05, Table 3). Based on the logistic regression analysis, a lower PA lever (> 144.5 vs ≤144.5 mg/L; OR, 6.514; 95% CI, 3.410–12.443), later corticosteroid therapy (≥4.5 vs < 4.5 d; OR, 2.333; 95% CI, 1.298–4.195), a higher CRP level (< 12.27 vs ≥12.27 mg/L; OR, 5.409; 95% CI, 3.041–9.622), and a higher LDH level (< 462.65 vs ≥462.65 U/L; OR, 4.377; 95% CI, 2.533–7.565) were each independently associated with BMP formation (Table 3).

**Table 3:** Logistic regression analysis after assigning risk factors for BMP formation

Variable (assignment)	Wald χ2	P	OR (95% CI)
CRP (< 12.27 mg/L = 0 ≥ 12.27 mg/L = 1)	33.000	0.000	5.409 (3.041–9.622)
LDH (< 462.65 U/L = 0 ≥ 462.65 U/L = 1)	27.983	0.000	4.377 (2.533–7.565)
PA (> 144.5 mg/L = 0 ≤ 144.5 mg/L = 1)	32.210	0.000	6.514 (3.410–12.443)
corticosteroids (< 4.5 d = 0 ≥ 4.5 d = 1)	8.018	0.005	2.333 (1.298–4.195)

Corticosteroids, use corticosteroids for the first few days

*BMP* bronchial mucus plug; *CI* confidence interval; *CRP* C reactive protein; *LDH* lactate dehydrogenase; *OR* odds ratio; *PA* prealbumin

### Percentage of mucous plug patients in the MPP scoring groups

**Fig. 5:**
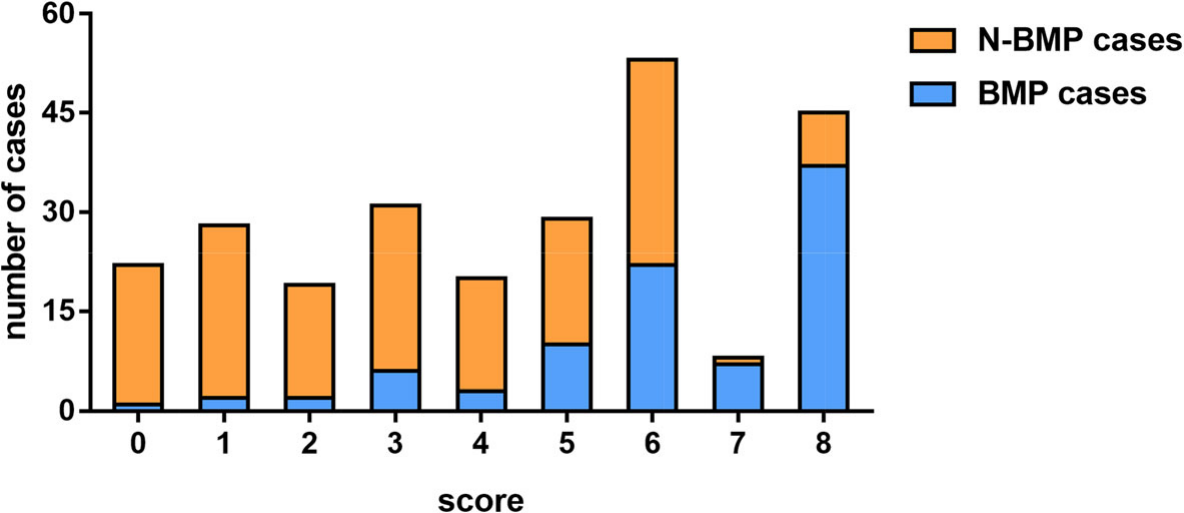
Correspondence between the scoring model and the formation of mucus plugs. According to the score, 255 children with *Mycoplasma pneumoniae* pneumonia (MPP) were divided into a high-risk group (7–8 points), a middle-risk group (4–6 points), and a low-risk group (0–3 points). Among them, the high-risk group had 44 [83.02%] mucus plugs out of 53; the middle-risk group had 35 [34.3%] mucus plugs out of 102; and the low-risk group had 11[11%] mucus plugs out of 100.

The independent risk factors were scored according to their OR value. The time of corticosteroids application ≥4.5 days was one point; a CRP ≥12.27 mg/L and an LDH ≥462.65 U/L was two points each; and a PA ≤144.5 mg/L was three points. According to the scores, the MPP patients were divided into a high-risk group (7–8 points), a middle-risk group (4–6 points), and a low-risk group (0–3 points). Among them, 53 cases were in the high-risk group and 44 cases (83.02%) were caused by mucus plugs. There were 102 cases in the middle-risk group, 35 cases (34.3%) with mucus plugs, 100 cases in the low-risk group, and 11 cases (11%) with mucus plugs (Fig. 5).

## Discussion

In recent years, with the increase in the incidence of MPP and the resistance to macrolide antibiotics, the incidence of refractory and severe MPP has increased. Studies have shown that BMP may become an important factor in the difficulty of MPP treatment [12]. The results of this study suggest that clinical variables, including a lower PA level (≤144.5 mg/L), later corticosteroid therapy (≥4.5 d), a higher CRP level (≥12.27 mg/L), and a higher LDH level (≥462.65 U/L) were significantly associated with presence of BMP in children with MPP.

Xu Q et al. found that CRP, LDH, age, and fever duration were associated with the formation of BMPs in children with refractory *Mycoplasma pneumoniae* pneumonia (RMPP) [10]. Xu X et al. did not find a significant difference in serum LDH and CRP levels between the children with and without BMPs [13]. It was found in this study that a CRP ≥12.27 mg/L and an LDH ≥462.65 were associated with BMP formation in children with MPP. This is related to the body’s excessive immune inflammatory response that leads to a numerous inflammatory factors. These inflammation factors further lead to serious airway mucosal damage, ciliary clearance dysfunction, and epithelial cell shedding, eventually forming a mucus plug to block the airway.

In contrast to previous studies, this study showed that the PA level had a higher predictive value. A PA ≤144.5 mg/L was associated with BMP formation in children with MPP. PA is a negative acute phase protein synthesized by the liver and is a non-specific host defense substance. Therefore, in an acute infection, the PA serum level can be rapidly reduced. Previous studies have found that PA is associated with the severity and prognosis of many diseases [13]. Shen et al. conducted a retrospective analysis of 174 children with community-acquired pneumonia (CAP) and found that the sensitivity of PA to diagnose CAP was higher than that of other inflammation indicators, which is an independent protective factor for children with CAP. Combined with CRP, PA can effectively improve the diagnostic efficiency of children’s CAP and assess the severity of pneumonia [14]. Research by Wang et al. showed that the PA level can reflect the severity of severe MPP, suggesting that the PA level may become an objective indicator for predicting the progress of severe MPP [15].

In addition, it was found that earlier use of corticosteroid (CS) therapy can reduce the formation of BMP in children with MPP. The optimal threshold was less than 4.5 days. CS therapy has a direct inhibitory effect on many inflammatory cells, which can inhibit neutrophil apoptosis, promote eosinophil apoptosis, and reduce the number of mast cells in the airway [16–18]. CS therapy can also inhibit inflammatory factors, improve clinical symptoms, reduce airway microvascular leakage, and reduce BMP production [19, 20].

In this study, the predictive values of the various risk factors for mucus plug formation were different, and they were assigned a predictive value according to their OR value. Among them, a PA ≤144.5 mg/L was three points; a CRP ≥12.27 mg/L and an LDH ≥462.65 U/L was two points each; and a time of CS therapy application of ≥4.5 d was one point. Therefore, children who met the above indicators obtained the highest score of 8 points. According to the scores, MPP patients with 7–8 points belonged to the high-risk group for BMP formation. For example, an MPP child with a PA level of ≤144.5 mg/L, CS therapy after 4.5 d, a CRP level of ≥12.27 mg/L, and an LDH level of ≥462.65 U/L strongly indicated the presence of a BMP.

This study had some limitations. First, laboratory samples were not collected for the same period as the presence of disease, which produced a bias. Second, a prospective study is required to further confirm the reliability for this retrospective study. Third, with the limited number of cases, it was difficult to make the number of cases in the two groups similar.

## Conclusions

PA level, timing of CS therapy use (use in the first few days), CRP level, and LDH level were independent risk factors for MPP mucus plug formation. According to the scoring system used in this study, the higher the score of children with MPP, the higher the risk of forming BMP. The scoring system does have the potential to be used for the identification of BMP in children with MPP, thereby contributing to a rational therapeutic choice.

## Data Availability

The datasets used and/or analysed during the current study are available from the corresponding author on reasonable request.

## Abbreviations

ALT: Alanine aminotransferase
AST: Aspartate aminotransferase
AUC: Area under curve
BMP: Bronchial mucus plugs
CAP: Community-acquired pneumonia
CD: Cluster of differentiation
CI: Confidence interval
CK-MB: Creatine kinase isoenzyme
CRP: C reactive protein
CS: Corticosteroid
CT: Computed tomography
DD: D-dimer
EOS: Eosinophil count
EPO: Eosinophil peroxidase
IgA: Immunoglobulin A
IgG: Immunoglobulin G
LDH: Lactate dehydrogenase
MPP: *Mycoplasma pneumoniae* pneumonia
NPA: Nasopharyngeal aspirates
OR: Odds ratio
PA: Prealbumin
ROC: Receiver operating characteristic
WBC: White blood cells
